# Bone Marrow-Derived Matrix Metalloproteinase-9 Is Associated with Fibrous Adhesion Formation after Murine Flexor Tendon Injury

**DOI:** 10.1371/journal.pone.0040602

**Published:** 2012-07-11

**Authors:** Alayna E. Loiselle, Benjamin J. Frisch, Matthew Wolenski, Justin A. Jacobson, Laura M. Calvi, Edward M. Schwarz, Hani A. Awad, Regis J. O’Keefe

**Affiliations:** 1 Center for Musculoskeletal Research, University of Rochester, Rochester, New York, United States of America; 2 Endocrine Division, Department of Medicine, University of Rochester School of Medicine and Dentistry, Rochester, New York, United States of America; 3 Department of Biomedical Engineering, University of Rochester, Rochester, New York, United States of America; University of Liverpool, United Kingdom

## Abstract

The pathogenesis of adhesions following primary tendon repair is poorly understood, but is thought to involve dysregulation of matrix metalloproteinases (Mmps). We have previously demonstrated that *Mmp9* gene expression is increased during the inflammatory phase following murine flexor digitorum (FDL) tendon repair in association with increased adhesions. To further investigate the role of *Mmp9*, the cellular, molecular, and biomechanical features of healing were examined in WT and Mmp9^−/−^ mice using the FDL tendon repair model. Adhesions persisted in WT, but were reduced in Mmp9^−/−^ mice by 21 days without any decrease in strength. Deletion of *Mmp9* resulted in accelerated expression of neo-tendon associated genes, *Gdf5* and *Smad8,* and delayed expression of *collagen I* and collagen *III*. Furthermore, WT bone marrow cells (GFP^+^) migrated specifically to the tendon repair site. Transplanting myeloablated Mmp9^−/−^ mice with WT marrow cells resulted in greater adhesions than observed in Mmp9^−/−^ mice and similar to those seen in WT mice. These studies show that *Mmp9* is primarily derived from bone marrow cells that migrate to the repair site, and mediates adhesion formation in injured tendons. *Mmp9* is a potential target to limit adhesion formation in tendon healing.

## Introduction

While flexor tendon repair following traumatic transection restores the continuity of the tendon tissue, the healing process is frequently complicated by scarring and adhesion formation [1], [2]. The scar tissue formed in association with the injury and repair process impedes the ability of low friction gliding of the tendon through the tendon synovial sheath. Clinical interventions such as controlled passive motion (CPM) [3], [4] therapy protocols have reduced but not eliminated adhesion formation. The lack of motion of one of the fingers interferes substantially with the function of the entire hand. Although flexor tendon injuries are common and simple to surgically repair, the injury is associated with significant disability and physical impairment.

Tendon healing is a complex process that involves the coordinated interaction of numerous cell types. Following injury, an initial inflammatory reaction including hematoma formation occurs within 24 hours. Inflammatory cells such as neutrophils and macrophages migrate to the repair site, and engulf cellular debris [5], while tenocytes initiate synthesis of tendon extracellular matrix. The proliferative phase of healing is marked by the rapid deposition of an abundant but disorganized collagen matrix that is composed primarily of type III collagen. The newly synthesized collagen matrix re-establishes continuity of the tendon and provides some mechanical strength. During this phase of healing the tendon ends adjacent to the injury site undergo catabolism [5] and are replaced with granulation tissue. This expands the initial zone of injury and increases the total volume of tissue involved in the injury and repair process. While the exuberant injury response ensures healing and reconstitutes tendon strength, it increases scarring. The tendon becomes adherent to adjacent structures and the impaired tendon gliding ultimately decreases the overall finger arc of motion. The proliferative phase begins about four days after repair, and lasts for four weeks in human tendon healing [6]. In this murine model, the proliferative phases occurs until about 14 days post-repair [7]. Over time the repair tissue undergoes remodeling, synthesis of type I collagen is increased, and scar tissue is replaced with normal tendon. Thus, minimizing the formation of scar tissue while maximizing tendon strength is a goal of successful flexor tendon repair [1].

A potential strategy to decrease adhesions involves reduction of the initial zone of injury by limiting the catabolism of tendon tissues adjacent to the injury site. Type I Collagen comprises between 65–85% of the normal tendon [8], [9], [10], [11]. Matrix Metalloproteinase-9 (*Mmp9*; Gelatinase B) is involved in the early neo-vascularization that occurs at sites of injury, and may be involved in degradation of the extracellular collagen matrix [12], [13]. In human tendon tissue derived from patients with degenerative patellar tendinopathy, rotator cuff tendinitis, and Achilles tendon rupture, *Mmp9* up-regulation is observed [14], [15], [16]. *Mmp9* is implicated in scar formation in the spinal cord [17] and the lung [18]. In an experimental model of intrasynovial murine flexor tendon repair, we recently demonstrated elevated *Mmp9* expression during the early-inflammatory period of tendon healing [7]. This is the period of maximal tissue catabolism and extension of the zone of injury.

*Mmp9* has been implicated in scarring following myocardial injury, central nervous system injury, lung injury, liver injury, and in kidney disease [19], [20], [21], [22], [23], [24], [25], [26]. Furthermore, *Mmp9* gene deletion results in reduced interstitial fibrotic lesions in mice with obstructive nephropathy, inhibits allergen induced lung fibrosis, and attenuates fibrosis in the liver following injury [20], [25], [27]. A polymorphism that results in decreased expression of the *Mmp9* gene is associated with decreased scar formation and blindness in patients with trachoma eye infection [28]. Collectively, these observations suggest that *Mmp9* is directly involved in scar formation during adult tissue repair.

The source of *Mmp9* in injury and repair processes can either be local stromal cells, bone marrow cells, or systemically derived macrophages or mesenchymal precursors recruited to the injury site [29], [30], [31], [32]. Inflammatory cytokines induced by injury or other conditions stimulate *Mmp9* expression in tissue fibroblasts [32]. For example, neural stem cells in a hypoxic environment have a five-fold up-regulation of *Mmp9* [33]. Astrocytes, kidney mesangial cells, and cardiac, synovial and dermal fibroblasts also have been shown to express *Mmp9* [22], [31], [34], [35], [36], [37]. Macrophages recruited to the injured tissues have been shown to be a major source of *Mmp9* expression in central nervous system injury, liver fibrosis, and pulmonary disease [19], [27], [38]. Bone marrow-derived hematopoietic cells expressing *Mmp9* are recruited to skin cancers where they modulate tumor invasiveness in a mouse model [12].

This study defines the role of *Mmp9* during tendon healing and in the process of adhesion formation using a murine intrasynovial flexor digitorum longus tendon injury and repair model [7], [39]. Tendon injuries heal in Mmp9^−/−^ mice with decreased adhesions, more robust tissue remodeling and with accelerated expression of molecular markers of tendon differentiation. Transplantation of bone marrow from Mmp9^−/−^ donor mice into irradiated WT (wild type) recipients and vice versa, demonstrates that *Mmp9* expression during tendon repair is derived from cells that originate in the bone marrow and migrate to the injury site. This work identifies a novel molecular and cellular target for therapeutic intervention to preempt formation of fibrous adhesions after flexor tendon injury.

## Results

### Loss of Mmp9 Alters Gene Expression during Flexor Tendon Healing

An intrasynovial FDL tendon repair model [7], [39] was used in Mmp9^−/−^ and WT mice to examine the role of *Mmp9* in tendon healing. Prior experiments in this model have shown peaks of *Mmp9* and *Col3* expression during the early-inflammatory phase of healing (seven-10 days), followed by a shift to *Col1* expression (14 days), and induction of *Mmp2* at the initiation of the remodeling phase of tendon healing when adhesion formation is highest (21 days). The expression of the tendon-associated transcription factor *Smad8* [40], [41] and tendon associated growth factor *Gdf5* are both markedly up-regulated between 28 and 35 days. This pattern of gene expression is associated with a reduction in the gliding coefficient (a biomechanical measure of adhesions), and a transition to tissue morphology that more closely resembles normal tendon tissue rather than scar tissue [7].

**Figure 1:**
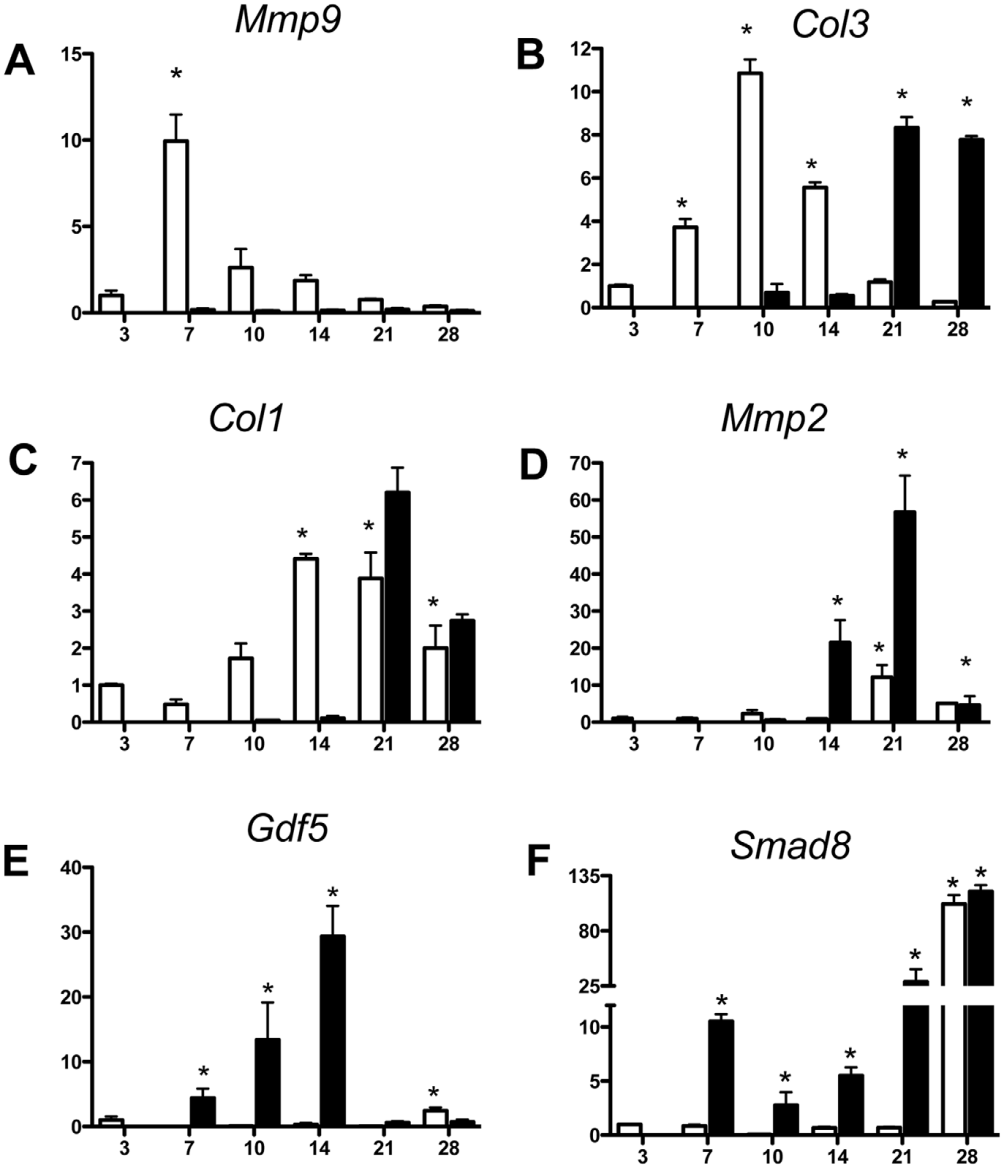
Early expression of neo-tendon associated genes during flexor tendon healing in Mmp9 ^−**/**−^ **mice.** Gene expression of (A) *Mmp9* (B) *Col3a1*, (C) *Col1a1*, (D) *Mmp2*, (E) *Gdf5*, and (F) *Smad8* in FDL tendon repair tissue over time up to 28 days post-op. Total RNA was extracted and pooled from five tendon repairs per time-point and processed for real-time RT-PCR. Gene expression was standardized with the internal *β-actin* control and then normalized by the level of expression in day three WT FDL tendon repairs. Data presented as the mean fold induction (over WT day three repairs) ± SEM. * p<0.05 vs. WT day three tendon repair. White bars represent WT mice. Black bars represent Mmp9^−/−^ mice.

The expression of genes associated with the inflammatory, proliferative and remodeling phases of healing in FDL injured tendons of WT and Mmp9^−/−^ mice were analyzed by real-time RT-PCR and normalized to their expression level in day three repairs in WT mice (Figure 1). Peak expression of *Cox-2*, an important mediator of inflammation, occurred at three days post-repair in WT and Mmp9^−/−^ tendons, however, expression was not significantly different between groups at this time (p>0.05). *Cox-2* was not differentially expressed between WT and Mmp9^−/−^ at any time during healing, with slight, but in-significant decreases after day three (Figure S1). *Mmp9* was not expressed in reparative tissues in Mmp9^−/−^ mice, but its expression in WT mice was similar to that observed in prior studies [7], with peak expression at seven days and a return to control levels by 14 days (Figure 1A). Type III Collagen (*Col3*), which is associated with granulation tissue, was increased at seven days post-repair in WT mice (3.2-fold), and had peak expression at 10 days post-repair (10.8-fold increase). *Col3* expression remained significantly elevated through 14 days (5.5-fold increase), after which time expression returned to baseline levels in WT mice (Figure 1B). In WT mice, peak expression of *Col1* was observed on day 14 post-repair (4.4-fold increase) and persisted through day 28 (2-fold increase; Figure 1C). In contrast, both *Col3* and *Col1* expressions were delayed in Mmp9^−/−^ mice. *Col3* expression was not significantly increased in Mmp9^−/−^ mice until day 21 (8.3-fold increase), but remained significantly elevated through day 28 (7.7-fold increase). The pattern of *Col1* expression in Mmp9^−/−^ mice was similar to that observed with *Col3*. Peak expression of *Col1* occurred on day 21 (6.2-fold increase), and remained elevated through day 28 (2.7-fold increase; Figure 1B&C).

*Mmp2* (Gelatinase A) expression is associated with the tissue remodeling phase of tendon healing when adhesions decrease [7]. *Mmp2* was expressed earlier and at increased levels in tendon repairs in Mmp9^−/−^ mice compared to WT mice (Figure 1D). *Mmp2* expression was increased beginning at day 14 post-repair (21-fold increase; Figure 1D) in Mmp9^−/−^ mice, and peak expression occurred on day 21 (56-fold increase). At day 28 *Mmp2* expression remained elevated (4.7-fold increase). In contrast, elevation of *Mmp2* expression was not observed until day 21 in WT mice (12-fold increase), although this also represented the point of maximal expression in these mice. At day 28, expression of *Mmp2* decreased relative to day 21, but remained elevated (5.1-fold increase) compared to day three WT controls.

The earlier and more robust expression of *Mmp2* in the tendon repairs of Mmp9^−/−^ mice suggests more vigorous and rapid tendon regeneration and remodeling. To further examine this possibility, the expression of genes associated with neo-tendon formation, *Gdf5* and *Smad8* were examined in tendon repair tissues in Mmp9^−/−^ and WT mice [41], [42], [43]. In healing tendons in WT mice, *Gdf5* and *Smad8* expression became elevated only late in the repair process (day 28) and following the peak expression of *Mmp2*. *Gdf5* was increased 2.5-fold and *Smad8* was increased 106-fold at 28 days following injury in WT mice. In contrast, the expression of these neo-tendon related genes was markedly accelerated in Mmp9^−/−^ mice. *Gdf5* was elevated by seven days post-repair (4.4-fold), and elevated expression persisted through day 14 (29.3 fold increase; Figure 1E). *Smad8* was also induced by seven days following tendon repair in Mmp9^−/−^ mice (10.5-fold increase; Figure 1F), and remained elevated through day 28, when a 119-fold increase was observed.

### Mmp9^−/−^ Tendons have a Reduced Zone of Injury

**Figure 2:**
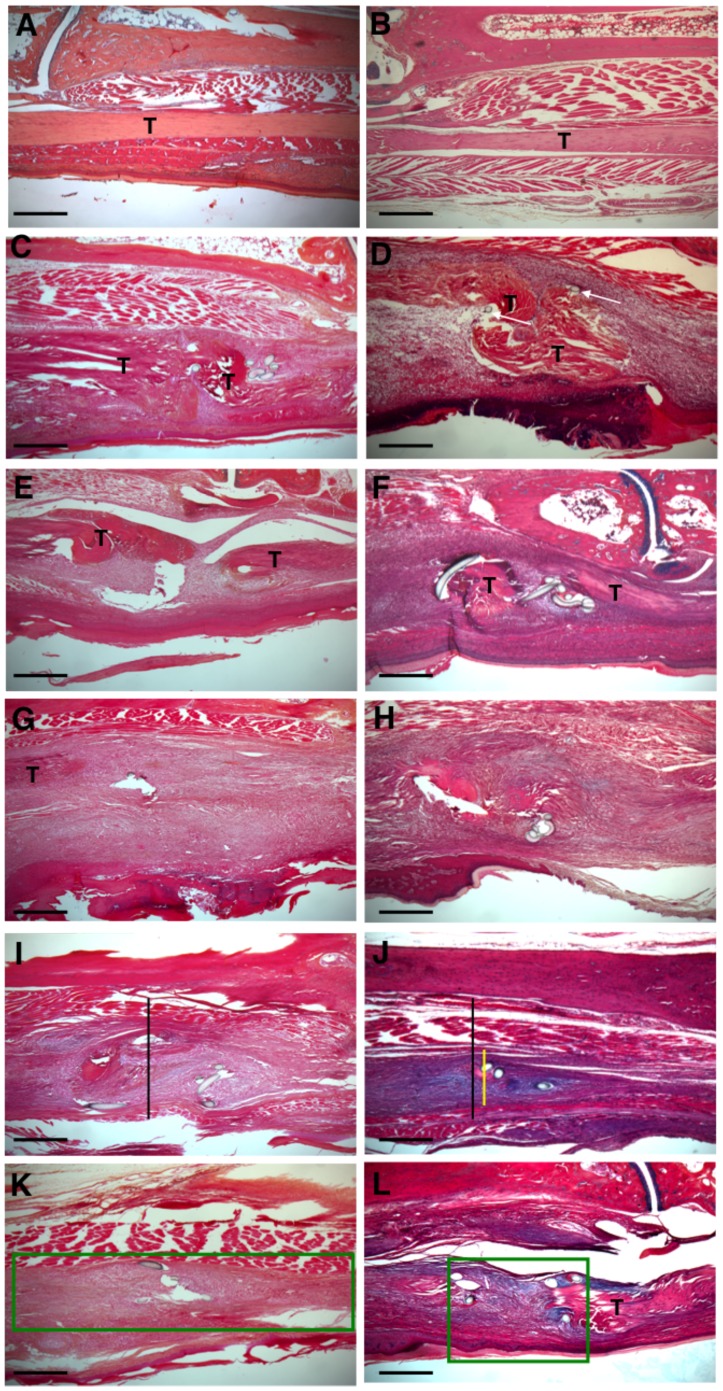
Decreased area of remodeling during tendon healing in Mmp9 ^−**/**−^ **mice.** Representative histological sections of sham control WT (A) and Mmp9^−/−^ (B) FDL Tendons. Repaired WT and Mmp9^−/−^ FDL Tendons at days 7 (C, D), 10 (E, F), 14 (G, H) 21 (I, K) and 28 (K, L). Sections were stained with Alcian Blue/Hematoxylin and Orange G. Of note is the fibroblastic granulation tissue that fills in the repair site between tendon ends (marked as T) and is progressively remodeled (green box) with collagen becoming oriented in a parallel fashion with the long axis of the tendon. Black bar represents width of WT tendon; yellow bar represents width of Mmp9^−/−^ tendon at Day 21. Scale bars represent 200 microns. 5X magnification.

Histology was used to assess changes in the structure and morphology of healing FDL tendons. There were no observable differences in the structure of sham control tendons between WT and Mmp9^−/−^ mice (Figure 2 A&B).

In WT mice at seven days post repair, a reactive, cellular granulation tissue developed adjacent to the tendon repair site and at the repair site (Figure 2C). At day 10 post-repair, granulation tissue was more cellular and the native collagen tissue adjacent to the repair site underwent catabolism and invasion by the reactive granulation tissue (Figure 2E). At 14 days the ends of the tendon were bridged at the repair site by granulation tissue and an external callus of thickened granulation tissue encased the repair site and adjacent tendon tissue (Figure 2G). At day 21, the granulation tissue reached maximal thickness. The tissue was composed of disorganized collagen fascicles, which replaced the tendon ends adjacent to the repair (Figure 2I). At day 28 remodeling of the tendon occurred with thinning of the granulation tissues that encased the tendon. At this time point extensive remodeling and replacement of the original tendon tissue was observed, with more organized tissue that was oriented parallel to the long axis of the tendon. The area of tendon replacement by the healing scar tissues comprised the entire zone of injury and is show with the green box (Figure 2K).

The tendon repair process in Mmp9^−/−^ mice had similarities and some unique features compared to WT repairs. While WT and Mmp9^−/−^ tendons healed with increased granulation tissue at the repair site by day seven (Figure 2C&D), the width of the granulation tissue was reduced at subsequent time points in Mmp9^−/−^ repairs (Days 10, 14, 21, and 28; Figures 2F, 2H, 2J, and 2L). The difference in the width of the reparative tissue is apparent in the photomicrographs following repair in the WT and Mmp9^−/−^ mice. The black bar (Figure 2I and 2J) shows the width of the granulation tissue in WT mice compared to the width of the granulation tissue in the Mmp9^−/−^ mice (yellow bar; Figure 2J). Another distinguishing feature is that the degree of catabolism of native tendon tissue adjacent to the repair site is reduced. This results in a reduction of the extent of the zone of injury. This is readily apparent on photomicrographs of WT and Mmp9^−/−^ repair tissues at day 28. The green boxes show the extent of the zone of injury and area of native tendon replacement and remodeling in WT mice (Figure 2K) and Mmp9^−/−^ mice (Figure 2L). Altogether, the molecular and histological data show that Mmp9^−/−^ mice undergo tendon healing with less catabolism and extension of the zone of injury and have a concomitant acceleration of the reduction and remodeling of granulation tissues.

### Adhesions are Remodeled Earlier in Mmp9^−/−^ Flexor Tendons

**Figure 3:**
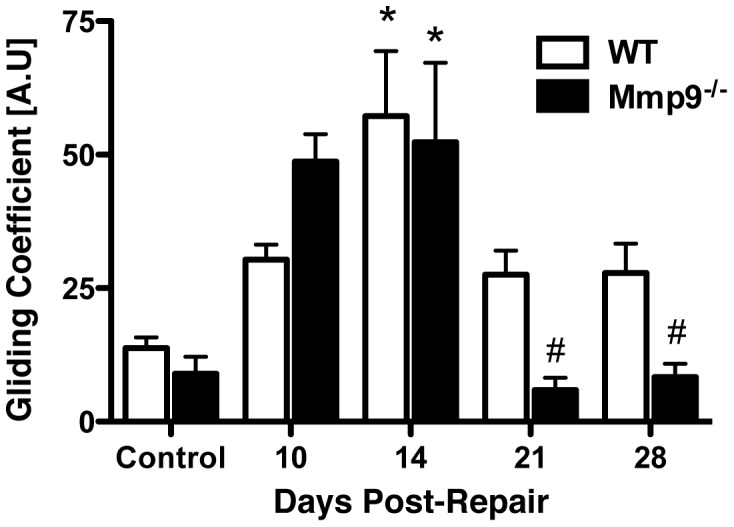
Earlier resolution of fibrous adhesions in Mmp9 ^−**/**−^ **tendon repairs.** Gliding coefficient of WT and Mmp9^−/−^ mouse FDL tendon repairs over time up to 28 days post-repair. Repair data is compared to sham control tendons (mean ± SEM). (*) Indicates significant difference versus respective control, while (**#**) indicates significant difference versus Day 14 Mmp9^−/−^ repair.

The gliding coefficient is derived from a biomechanical measurement of metatarsal-phalangeal (MTP) joint range of motion following a series of applied loads to the tendon [7], [39]. An elevated gliding coefficient is associated with a higher force required for a given degree of flexion, and therefore is an index of scar and adhesion formation [7], [39]. The baseline WT gliding coefficient was not statistically different than the controls from Mmp9^−/−^ mice. The gliding coefficient was increased at day 10 and day 14 in the repairs from both WT and Mmp9^−/−^ mice. However, there was no significant difference in the gliding coefficients at these time points, and in both WT and Mmp9^−/−^ mice maximal adhesions occurred at 14 days (WT, 4.1-fold; Mmp9^−/−^ mice, 5.8-fold; Figure 3). A significant difference was observed beginning at 21 days following repair. In Mmp9^−/−^ mice the gliding coefficient had returned to baseline levels, consistent with tendon remodeling. In contrast, the gliding coefficient remained elevated in the repairs of the WT mice at 21 days (2-fold). Similar differences were observed at 28 days. Thus the biomechanical measure of the tissue adhesions further confirms that Mmp9^−/−^ mice undergo tendon repair with reduced scar formation.

### Strength and Stiffness are Maintained in the Repairs of Mmp9^−/−^ Mice

Biomechanical measurement of the maximum tensile load at failure of repaired and control tendons were determined in both the WT and the Mmp9^−/−^ mice (Table 1). Control tendons in the WT and Mmp9^−/−^ mice had a similar load at failure. Following repair, the maximum load at failure was markedly decreased after 14 days of healing in both WT mice (8.8% of control) and Mmp9^−/−^ mice (16.4% of control) (Table 1). The maximal load at failure progressively increased as the repair progressed. However, by 28 days, repaired tendons in WT mice had reached only 25% of the maximum load at failure of un-operated WT tendon, while repaired tendons in Mmp9^−/−^ mice had regained only 31% of the maximum load at failure of un-operated Mmp9^−/−^ tendon.

**Table 1:** Maximum load at failure [N] of WT and Mmp9^−/−^ mouse FDL tendon repairs over time up to 28 days.

Days Post-Repair	WT	Mmp9^−/−^
Control	10.1±0.39	9.2±1.01
10	0.89±0.31*	1.51±0.27*
14	0.89±0.24*	2.29±0.22*
21	2.15±0.30*	2.34±0.21*
28	2.52±0.34*	2.88±0.31*

Repair data is compared to sham control tendons (mean ± SEM), (*) indicates p<0.05 compared to respective control.

The stiffness (the slope of the linear region of the force displacement curve) of the control and repaired tendons in WT mice and Mmp9^−/−^ mice were also measured (Table 2). Similar stiffness was observed in the control (un-operated) tendons from WT mice and Mmp9^−/−^ mice (Table 2). As with measurements of strength, the stiffness of the repaired tendons also increased with the time of healing. The stiffness of the tendon repairs of the WT and Mmp9^−/−^ mice were similar at each time point. At 14 days, Mmp9^−/−^ repairs were 36% stiffer than WT, although this difference was not significant (p = 0.13). At 28 days repaired tendons in the WT mice and Mmp9^−/−^ mice regained 44.9% and 45.9% of their stiffness, respectively (Table 2).

**Table 2:** Stiffness [N/mm] of WT and Mmp9^−/−^mouse FDL tendon repairs over time up to 28 days.

Days Post-Repair	WT	Mmp9^−/−^
Control	5.65±0.33	4.88±0.17
10	1.63±0.15*	1.19±0.02*
14	1.46±0.17*	2.00±0.21*
21	1.89±0.26*	2.23±0.18*
28	2.54±0.26*	2.59±0.19*

Repair data is compared to sham control tendons (mean ± SEM), (*) indicates p<0.05 compared to respective control.

### Bone Marrow Derived Cells Migrate to the Flexor Tendon Repair Site

To determine the ability of bone marrow-derived cells to migrate to the flexor tendon repair site, bone marrow mononuclear cells from C57B6/J GFP^+^ transgenic mice were transplanted into WT C57B6/J mice following conditioning whole body irradiation. After allowing the transplanted cells to engraft, the mice underwent flexor tendon transection and repair.

**Figure 4:**
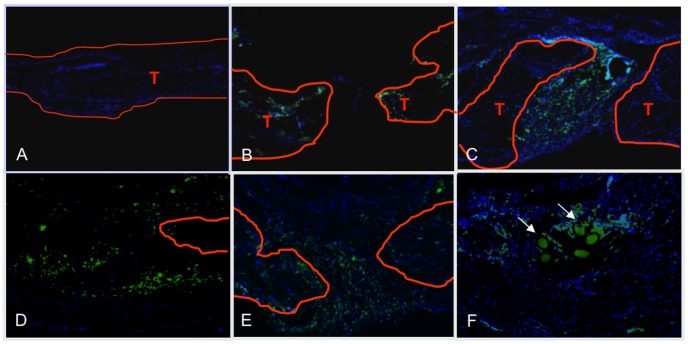
Bone marrow cells migrate specifically to the FDL repair site in vivo. Representative sections of repaired flexor tendons from C57Bl6/J mice that were myeloablated, and reconstituted with bone marrow from GFP transgenic mice. Tendons were repaired after bone marrow cells had engrafted, and tissues were harvested between three and 28 days post-repair. Sections were counterstained with the nuclear dye DAPI (blue) and bone marrow derived cells were identified based on the expression of GFP. Contralateral sham controls (A) did not have any GFP expressing cells indicating a lack of bone marrow cells in un-injured tendon, while bone marrow derived cells are present at the FDL repair site at (B) three, (C) seven, (D) 14, (E) 21, and (F) 28 days post-repair. Tendon tissue is outlined in orange and marked as ‘T’. All images are 10x magnification. Scale bars represent 200 microns.

The presence of bone marrow-derived cells at the repair site was determined by examining histological sections of healing tendon with fluorescent imaging for the expression of GFP. GFP^+^ cells were not observed in un-injured contralateral sham control tendons (Figure 4A). However, GFP^+^ cells were observed in the healing tendon tissues where they localized to the reparative granulation tissues. GFP^+^ cells were present three days after repair (Figure 4B) and increased numbers were observed by seven days (Figure 4C). GFP^+^ bone marrow-derived cells remained abundant at 14 days following repair and they were localized to the healing granulation tissue (Figure 4D). By day 21, GFP^+^ bone marrow derived cells were diffusely located throughout the granulation tissue and in areas of remodeled tendon tissue (Figure 4E). At 28 days post-repair the remaining GFP+ cells were present in the area of tendon remodeling (Figure 4F).

### Mmp9 is Expressed by Bone Marrow-derived Cells during Flexor Tendon Healing

In order to determine if bone marrow-derived cells migrating to the injury site provide local *Mmp9* expression in tendon repair, reciprocal bone marrow transplants were conducted in which WT mice received Mmp9^−/−^ bone marrow transplantation, and Mmp9^−/−^ mice received WT bone marrow transplantation.

**Figure 5:**
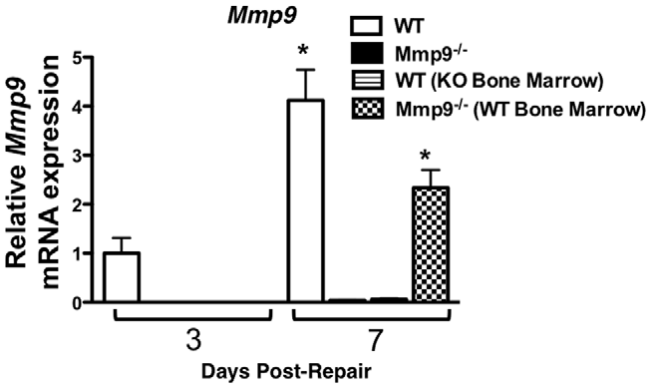
*Mmp9* is not expressed in healing tendons from WT mice with Mmp9 ^−**/**−^ **bone marrow.** Real-time RT-PCR for *Mmp9* expression in WT, Mmp9^−/−^ (KO), WT mice transplanted with bone marrow from an Mmp9^−/−^ donor (WT [KO Bone Marrow]), Mmp9^−/−^ mice transplanted with bone marrow from a WT donor (KO [WT Bone Marrow]) in repaired flexor tendons at three and seven days post-repair. Expression is normalized to *β-actin*, and compared day three expression in WT mice. Data are presented as mean ± SEM. (*) Indicates a significant difference compared to WT day three expression.

The expression of *Mmp9* was examined by real time RT-PCR in tissue extracted from the healing tendons of WT mice; WT mice following transplantation with Mmp9^−/−^ bone marrow cells; Mmp9^−**/**−^ mice; and Mmp9^−/−^ mice following transplantation with WT bone marrow cells (Figure 5A). Since we demonstrated earlier (Figure1A) that during tendon repair *Mmp9* expression peaks at day seven post injury, tissues from the repaired tendons were harvested at three and seven days post-repair and analyzed by real-time RT-PCR for expression of *Mmp9*. WT mice had *Mmp9* expression measured at three days following repair and this level of expression was used as a baseline level of normalized expression (1.0±0.54). *Mmp9* expression increased 4.1-fold at day seven in the WT mice. In contrast, WT mice with transplantation of Mmp9^−/−^ bone marrow cells did not have measurable levels of expression of *Mmp9* at day three and had minimal expression at day seven following repair (0.059±0.03; p<0.05, compared to the expression level in day three post-repair WT tendons). As expected, repairs from Mmp9^−/−^ mice lacked *Mmp9* gene expression. However, in Mmp9^−/−^ mice transplanted with bone marrow cells from WT mice, no detectable *Mmp9* expression was observed three days following repair, whereas a 2.3-fold increase (p<0.05; compared to WT day three expression) was observed at seven days after tendon repair (Figure 5). Collectively, these data show that *Mmp9* expression during tendon repair is dependent upon migration of bone marrow-derived cells to the injury site.

To determine the influence of host cells on bone marrow transplantation, the level of *Mmp9* expression in bone marrow from WT mice receiving Mmp9^−/−^ bone marrow, and Mmp9^−/−^ mice receiving WT bone marrow were analyzed. Mmp9^−/−^ mice with WT bone marrow had a significant 18.5-fold induction (p = 0.001) of *Mmp9* expression relative to WT mice with Mmp9^−/−^ bone marrow at seven days post-repair (Figure S2). There was a 2.84-fold increase in *Mmp9* expression in bone marrow from WT mice with Mmp9^−/−^ bone marrow, relative to *Mmp9* expression in tendon tissue in these mice, suggesting the presence of some host WT cells in the bone marrow of WT mice with Mmp9^−/−^ bone marrow.

### *Mmp9* Deficient Bone Marrow Cells are Associated with Decreased Tendon Adhesions

Quantification of adhesions and biomechanical testing was done to determine the functional consequences of both bone marrow cell-specific deletion of *Mmp9*, and expression of *Mmp9* solely in bone marrow cells during flexor tendon healing.

**Figure 6:**
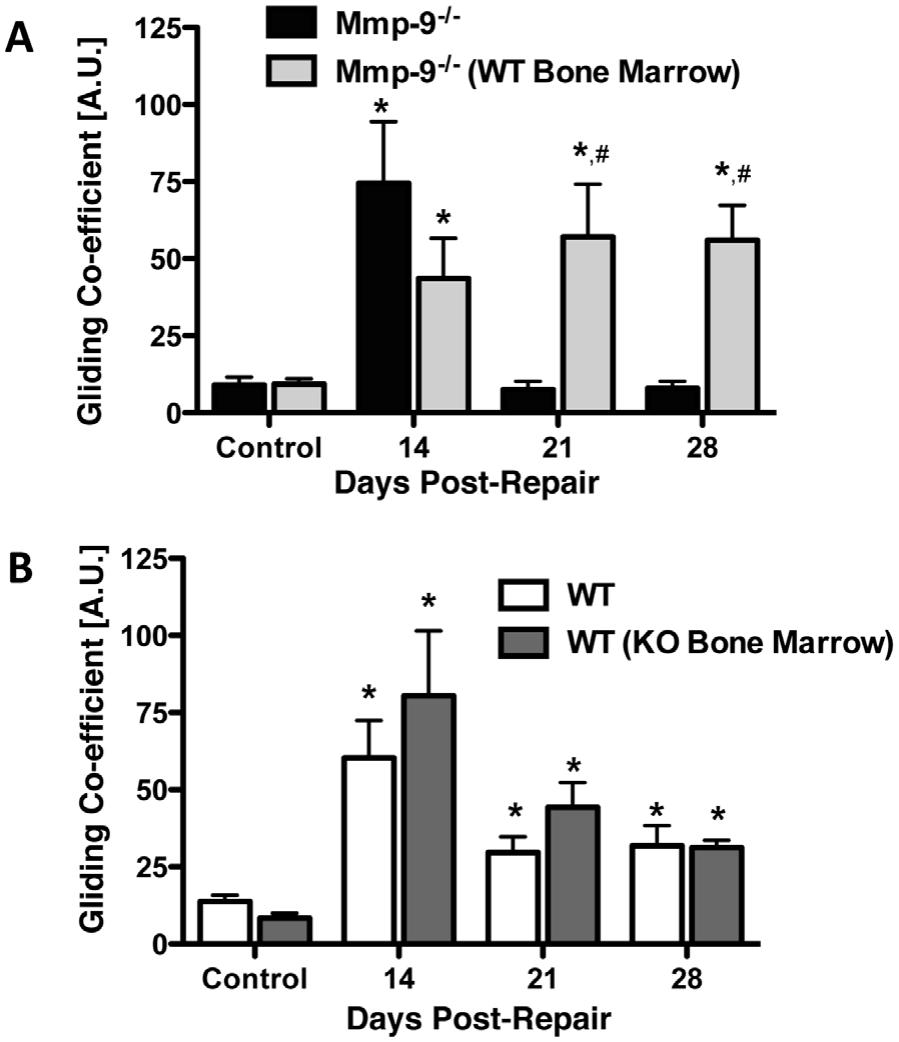
WT bone marrow in Mmp9 ^−**/**−^ **mice results in prolonged presence of adhesions during tendon healing.** Gliding coefficient based on range of applied load of FDL tendon repairs from [A] Mmp9^−/−^ mice, and Mmp9^−/−^ mice with WT bone marrow; [B] WT, and WT with Mmp9^−/−^ bone marrow between 14 and 28 days post-repair. (*) Indicates p<0.05 compared to respective control, # indicates p<0.05 compared to Mmp9^−/−^ repair at the same time-point. Data are presented as mean ± SEM.

To further define the role of bone marrow cell derived *Mmp9* in adhesion formation, MTP joint flexion tests were performed in Mmp9^−/−^ mice and in Mmp9^−/−^ mice following transplantation with WT bone marrow cells (Figure 6A). As previously shown, Mmp9^−/−^ mice had a reduced gliding coefficient during tendon healing, compared to WT controls. At 14 days following repair, the gliding coefficient was increased 8.3-fold, but was reduced to basal levels at day 21 and 28. In contrast, the transplantation of WT bone marrow cells into Mmp9^−/−^ mice resulted in an increased in gliding coefficient that mimicked the pattern of adhesions observed in WT mice undergoing tendon repair. Mmp9^−/−^ mice with WT bone marrow transplantation had a maximal gliding coefficient at 21 days (9-fold), but had sustained elevation of the gliding coefficient at 28 days (6-fold). The gliding coefficient was significantly elevated in the tendon repairs of the Mmp9^−/−^ mice with WT bone marrow transplantation compared to the tendon repairs of the Mmp9^−/−^ mice at 21 and 28 days. Therefore, rescue of *Mmp9* expression in bone marrow-derived cells is sufficient to reestablish tendon scarring after injury in *Mmp9*-deficient mice.

Adhesions were also measured in WT mice and in WT mice following transplantation with Mmp9^−/−^ bone marrow cells (Figure 6B). As previously shown, WT mice had a maximal increase in the gliding coefficient at 14 days after repair (approximately 4-fold) followed by a decrease at 21 (2.1-fold) and 28 (2.3-fold) days following repair. WT mice with transplanted Mmp9^−/−^ bone marrow cells similarly had maximal gliding coefficients at 14 days followed by a decrease at 21 and 28 days after repair. The gliding coefficients in the WT mice and in WT mice with Mmp9^−/−^ bone marrow cell transplants were not significantly different, suggesting that even very low levels of *Mmp9* produced locally in the healing tissues might be produced by cells other than migrating marrow cells. This could include the low levels of *Mmp9* produced by tenocytes, or alternatively, some other cell types. Another explanation is that other factors or non-marrow cell populations are also involved in the adhesion formation that occurs in this model. Finally, based upon low, but detectable expression of *Mmp9* in the bone marrow of WT mice with Mmp9^−/−^ bone marrow, there appear to be a small number residual WT bone marrow cells after conditioning radiation, which could provide sufficient *Mmp9* to result in scarring.

As a control for any possible effects of whole body irradiation and bone marrow transplantation, tendon repairs were performed and the gliding coefficient measured in transplant control mice (WT bone marrow cells transplanted in WT mice; and Mmp9^−/−^ bone marrow cells transplanted into Mmp9^−/−^ mice). There was no significant difference between the gliding coefficient in transplant controls compared to WT and Mmp9^−/−^ mice respectively at any time post-repair (data not shown).

### Bone Marrow-specific Loss of Mmp9 does Not Decrease the Maximum Load at Failure

Following adhesion testing, the tensile properties of repaired tendons were measured. There was no difference in the basal maximum load at failure of tendons from mice that received bone marrow transplants or un-transplanted WT mice and Mmp9^−/−^ mice (Table 3). After repair, the maximum load at failure was significantly lower than controls in all groups, but the maximum load at failure of repairs from Mmp9^−/−^ mice with WT bone marrow cells was not significantly different than those from WT mice with Mmp9^−/−^ bone marrow at any time. On day 28, tendons from WT mice with Mmp9^−/−^ bone marrow regained 22% of the maximum load at failure of control tendons, while Mmp9^−/−^ mice with WT bone marrow transplantation regained 19% of load to failure in control tendons (Table 3).

**Table 3:** Maximum load at failure of flexor tendon repairs following reciprocal bone marrow transplants between WT and Mmp9^−/−^ mice to result in WT mice with Mmp9^−/−^ bone marrow, and Mmp9^−/−^ mice with WT bone marrow.

Days Post-Repair	WT Mice/Mmp9^−/−^ bone marrow	Mmp9^−/−^ Mice/WT bone marrow
Control	9.73±1.64	9.93±2.04
14	2.06±0.17*	1.47±0.19*
21	2.01±0.30*	1.90±0.22*
28	2.15±0.22*	2.51±0.42*

Data are ± SEM, (*) indicates p<0.05 compared to respective control.

The stiffness of repaired tendons from these groups of mice was also analyzed. There was no significant difference between control specimens for any of the groups. At 14 days post-repair, the stiffness of all groups was significantly decreased compared to all controls, and stiffness of the repaired tendons remained significantly decreased compared to controls in all groups through 28 days post-repair. At day 28 post-repair, the stiffness of tendons from Mmp9^−/−^ mice with WT bone marrow transplantation was significantly decreased (p<0.05) compared to WT mice with Mmp9^−/−^ bone marrow transplantation (Table 4).

**Table 4:** Stiffness of flexor tendon repairs following reciprocal bone marrow transplants between WT and Mmp9^−/−^mice to result in WT mice with Mmp9^−/−^ bone marrow, and Mmp9^−/−^ mice with WT bone marrow.

Days Post-Repair	WT Mice/Mmp9^−/−^ bone marrow	Mmp9^−/−^ Mice/WT bone marrow
Control	5.38±0.46	5.16±0.44
14	1.30±0.31*	1.72±0.27*
21	1.42±0.32*	1.37±0.19*
28	2.10±0.17*^,#^	1.43±0.22*

Data are ± SEM, (*) indicates p<0.05 compared to respective control, (#) indicates p<0.05 compared to Mmp9^−/−^ mice with WT bone marrow at the same time-point.

## Discussion

We show that complete loss of *Mmp9* during flexor tendon healing results in earlier remodeling of adhesions compared to WT mice, without loss of repaired tendon strength. Accelerated, and increased expression of *Mmp2*, *Gdf5* and *Smad8* suggest the remodeling phase of flexor tendon healing occurs earlier in Mmp9^−/−^ mice, resulting in the subsequent decrease in the gliding coefficient. Additionally, the contribution of bone marrow-derived cells to the flexor tendon healing process was demonstrated by presence of GFP-expressing cells in WT mice following bone marrow transplant of GFP^+^ cells. The functional role of *Mmp9* expression in bone marrow-derived cells was demonstrated by the increased adhesions that occurred in myeloablated Mmp9^−/−^ mice transplanted with WT bone marrow cells. The gliding coefficient was similar to that observed in tendon repairs of WT mice. Notably, the strength and stiffness of repairs in Mmp9^−/−^ mice were similar to those observed in WT mice. Thus, lack of *Mmp9* reduces tendon adhesions but does not result in a weaker repair tissue.

Based on the causal association between inflammation and adhesion formation [44], [45], previous work has altered the healing course with anti-inflammatory treatment [46], [47]. These studies have demonstrated that an attenuated inflammatory response can decrease adhesions, however, decreased adhesions are often accompanied by a decrease in the strength of the repair during the early phases of healing, rendering these tendons unsuitable for aggressive physical therapy, and prone to re-rupture [46], [47]. Virchenko *et al* have shown that an early decrease in force at failure resulted from Cox-2 inhibition [47], suggesting that altering the normal cascade of inflammatory events leads to a decrease in the force at failure. Furthermore, a greater proportion of untreated specimens had a higher histology score, corresponding a greater amount of mature tissue, suggesting a larger zone of injury in mice treated with a Cox-2 inhibitor. In contrast, our work shows that deletion of *Mmp9* results in reduced catabolism of native tendon and a development of a smaller zone of injury compared to WT repairs. However, there is no reduction in the maximum load at failure compared to tendon repairs in WT mice. This is an important distinction because a decrease in the maximum load at failure increases the likelihood of rupture. Our data indicate that targeted inhibition of early *Mmp9* expression may improve flexor tendon healing.

The robust remodeling of adhesions and the decreased gliding coefficient by day 21 in Mmp9^−/−^ mice suggests an accelerated remodeling phase. Prior work in our laboratory has shown that tendon remodeling is associated with elevated expression of *Mmp2* [7]. Elevated expression of *Mmp2* occurs earlier in Mmp9^−/−^ mice (14 days) compared to WT mice (21 days). *Mmp2* expression is increased immediately before a significant decrease in the gliding coefficient, suggesting an important role for *Mmp2* in scar remodeling. The reduced area of initial catabolism of tendon adjacent to the repair site in tendons from Mmp9^−/−^ mice suggests that while degradation of some native tissue is beneficial, too much catabolism results in a more abundant response with a prolonged period of increased adhesions. While lack of *Mmp9* results in a change in the kinetics of tendon remodeling, the overall mechanical strength of the tendon repair, a key component of tissue regeneration, is not reduced in the Mmp9^−/−^ mice. The return of tendon strength requires a balance between degradation, collagen formation, and tissue remodeling. Alteration in the expression of *Mmp9* changes the anabolic phase of healing, suggesting that *Mmp9* expression is a key regulator of subsequent tissue responses.

We show that bone marrow cells migrate to the flexor tendon repair site consistent with a prior report showing migration to the patella tendon repair site [29]. By day seven post-repair there is a large influx of cells that declines, but persists through day 28. The important role of inflammatory mediators or chemotactic signaling in this “homing” process is demonstrated by the complete absence of GFP+ cells in contralateral control tendons, indicating that BMSCs migrate specifically to the site of injury. Our findings differ somewhat from other models of bone marrow cell migration to the tendon repair site. In a murine partial patella tendon defect model there is a massive influx of GFP+ cells to the repair site within 24 hours [29], while we found very few bone marrow cells at three days post-repair. The rate of healing and the disruption in vascularization in a partial defect model may be different than that of a complete transection. The presence of intact tendon at the repair site may increase the early intrinsic phase of healing, or increase the release of chemotactic signals from the tendon tissue that recruits bone marrow cells to the repair site. Additionally, the synovial sheath surrounding the flexor tendon may be an additional barrier that bone marrow cells must cross before arriving at the intrasynovial repair site.

Expression of *Mmp9* specifically in cells derived from the bone marrow was achieved by bone marrow transplantation, while the functional consequences of bone marrow-specific *Mmp9* deletion were also shown. Loss of *Mmp9* in the bone marrow during flexor tendon healing resulted in earlier remodeling of adhesions, compared to those mice that express normal levels of *Mmp9* in the bone marrow, as well as in mice with bone marrow cell-specific *Mmp9* expression. Resolution of fibrous adhesions after flexor tendon injury did not occur to the same degree in mice with bone marrow cell-specific loss of *Mmp9* compared to healing in Mmp9^−/−^ mice. Based on the low-level expression of *Mmp9* transcripts in tendons from WT mice with Mmp9^−/−^ bone marrow, it appears that marrow cells are a major source of *Mmp9* during flexor tendon healing. It appears that some WT host cells persisted in the bone marrow and provided a slight contribution of *Mmp9* during flexor tendon healing. This would explain why there was not complete remodeling of adhesions to the extent of complete loss of *Mmp9*, and also why there was low, but detectable expression of *Mmp9* in tendons from WT mice with Mmp9^−/−^ bone marrow by real-time PCR.

Bone marrow-specific loss of *Mmp9* leads to earlier remodeling of adhesions, without a decrease in the strength of the repair. This study presents a novel mechanism of improved flexor tendon healing, by inhibition of *Mmp9*, which in the setting of tendon repair seems to be primarily produced by bone marrow-derived cells. Small molecule *Mmp9* inhibitors are currently in clinical trials for Multiple Sclerosis, Chronic Obstructive Pulmonary Disease and Prostate Cancer [38], [48], and may aid the pursuit of *Mmp9* inhibition during tendon healing. This work demonstrates that it is possible to regain gliding function of the flexor tendon sooner, without compromising the strength of the repair. One limitation of this study is that we have not directly assessed the in vivo gelatinolytic activity of *Mmp9* during flexor tendon healing. Identification of this activity would identify a novel target to improve flexor tendon healing.

## Methods

### Ethics Statement

All animal procedures were approved by the University Committee on Animal Research at the University of Rochester.

### Mouse Strain Information

All mice were acquired from Jackson Laboratories (Bar Harbor, ME). Six to eight week old female Mmp9^−/−^ (FVB.Cg-Mmp9^tm1Tvu^/J) [12] and WT (FVB/NJ) mice were used for tendon repair studies and bone marrow transplants. GFP transgenic (C57BL/6-Tg(UBC-GFP)30Scha/J) and C57BL/6 mice were used for initial bone marrow transplant studies.

### Murine Flexor Tendon Injury and Repair

Complete transection and repair of murine flexor tendons was conducted as previously described [7] Six to eight week old female mice (Jackson Laboratories, Bar Harbor, ME) were anesthetized by intraperitoneal injection of 4 mg/kg xylazine and 60 mg/kg ketamine. The distal flexor digitorum longus (FDL) tendon was exposed and two horizontal 8-0 nylon sutures in a modified Kessler pattern were placed in the intact tendon. The tendon was then transected between the sutures and repaired by approximating the transected ends using the suture. The tendon was also transected proximally along the tibia at the myotendinous junction to protect the repair. The skin was closed with running 4-0 nylon sutures. The mice were returned to their cages and allowed free active motion and weight bearing following recovery from anesthesia. Sham surgeries were performed on control groups. Identical anesthesia and exposure were performed. When the distal FDL tendon was isolated, two horizontal 8-0 sutures in a modified Kessler pattern were sutured through the tendon. This suture was then removed without transecting the tendon. However the proximal FDL tendon was released at the myotendinous junction as in the repair group. The skin was closed with running 4-0 nylon sutures.

### RNA Extraction and Real-Time RT-PCR

Tendons were harvested and RNA was extracted as previously described [7]. Briefly, five repaired tendons per time-point per experimental group were harvested and RNA was extracted using TRIzol (Invitrogen). cDNA was reverse transcribed using 1ug of RNA and the iScript cDNA synthesis kit (BioRad). Real-Time PCR was done using Absolute qPCR SYBR (ABgene) and gene specific primers (Table 5). Gene expression was normalized to *β-actin*, and expression on day three in WT tendons due the relative metabolic inactivity of un-injured tendons.

**Table 5:** Primer sequences for Real-time RT-PCR.

Gene	Forward (5′-3′)	Reverse (5′-3′)
*Col1a1*	gagcggagagtactggatcg	gcttcttttccttggggttc
*Col3a1*	gcccacagccttctacac	ccagggtcaccatttctc
*Gdf5*	ggcaaagcatcttcaaaagc	ccaacttcacgctgctgtta
*Smad8*	gcctacgcaagtgtgtcacca	aggctgagctgagggttgta
*Mmp2*	agatcttcttcttcaaggaccggtt	ggctcctcagtggcttggggta
*Mmp9*	tgaatcagctggcttttgtg	accttccagtaggggcaact
*β-actin*	agatgtggatcagcaagcag	gcgcaagttaggttttgtca

### Histological Analysis of Healing

Whole hind limbs containing repaired tendons were harvested between seven and 28 days post-repair as previously described [49]. The intact foot and tibia were harvested by disarticulating the hind limb at the knee. Briefly, the hind limbs were fixed in 10% formalin with the tibia at 90° relative to the foot, and then decalcified in 10% EDTA at 4°C for 28 days. The decalcified tissues were dehydrated and embedded in paraffin to preserve the anatomical relationship between the repaired tendon and surrounding tissues. Serial three-micron sagittal sections through the FDL tendon plane were then cut and stained with Alcian Blue/Hematoxylin and Orange G. Four specimens per group per time-point were used, with approximately five sections per specimen analyzed.

### Adhesion and Biomechanical Testing

Adhesion and biomechanical testing was completed as previously described [7], [49]. The hind limb was disarticulated at the knee, and the FDL tendon was released from the surrounding tissue proximal to the tarsal tunnel. The FDL tendon was secured between two pieces of tape using superglue. The limb was fixed in a custom apparatus, and a digital image was taken of the MTP joint in a neutral unloaded position with the digits extended. Loads were incrementally applied to the flexor tendon and the MTP joint range of motion relative to the unloaded position was measured from a digital image. MTP joint flexion angles were measured using ImageJ software (http://rsb.info.nih.gov/ij/), and plotted versus the applied loads. The gliding coefficient was determined by non-linear regression as a measure of resistance to MTP flexion to due impaired gliding.

Immediately following adhesion testing, the proximal aspect of the FDL tendon was released from the tarsal tunnel, the calcaneus and tibia were removed and the proximal end of the FDL was gripped in the Instron device as previously described [7], [49]. The distal bones of the foot were also secured in the Instron device (Instron 8841 DynaMight™ axial servohydraulic testing system, Instron Corporation, Norwood, MA). The tendon was tested in tension in displacement control at a rate of 30 mm/minute until failure. Force-displacement data were automatically logged and plotted and the maximum tensile force and stiffness were determined. N = 7–10 animals/group/time-point.

### Statistical Analysis

Biomechanical and Real-Time RT-PCR data were analyzed using a two-way analysis of variance (ANOVA) followed by Bonferroni’s multiple comparisons with a significance level of α = 0.05. p values are reported for significance compared to gene expression in WT day three samples; and biomechanical significance is reported compared to control tendons, except where otherwise stated.

### Bone Marrow Transplantation

C57BL/6 mice (Jackson Labs) were myeloablated and bone marrow transplantation was performed as previously described [50]. Briefly, an initial 5Gy whole body dose was given 24 hours prior to transplantation, and a second 5Gy dose was given two hours prior to transplantation. Whole bone marrow was aseptically isolated from mice expressing GFP in all tissues (C57BL/6-Tg (UBC-GFP) 30Scha/J, Jackson lab) and re-suspended in sterile PBS containing 2% FBS. Each mouse was given 5×10^5^ GFP+ cells via tail vein injection. Animals were preemptively given Sulfatrim antibiotic (Activas, Baltimore, MD) in the drinking water beginning seven days prior to irradiation, and were maintained on antibiotics for two weeks post-transplantation. Mice were allowed to recover and cells engrafted for two weeks post-transplant. At this time, standard flexor tendon repair was performed, and tendons healed for between three and 28 days. Tendons were harvested from the tarsal tunnel to the bifurcation at the digits, and placed in 4% Paraformaldehyde for one hour. Tissues were washed twice in PBS for 15 minutes, and placed in 15% sucrose for 1 hour until tissue was saturated. Tissues were then placed in 30% sucrose for 16 hours, and embedded in OCT media. Eight-10uM sections were cut using a cryostat. Sections were post-fixed with 0.2% Glutaraldehyde for 10 minutes, washed twice with PBS, counterstained and cover slipped using Vectashield Mounting Medium with DAPI (Vector Laboratories, Burlingame, CA) GFP was localized using fluorescent microscopy. N = 3 bone marrow transplant recipients/time-point.

WT and Mmp9^−/−^ bone marrow transplants were conducted in the same manner as described above, with tissues harvested for Real-time RT-PCR (N = 5/group/time-point), histology (N = 4/group/time-point; 5 sections/specimen), *in situ* hybridization, as well as biomechanical and adhesion testing (N = 7–10 group/time-point).

## Supporting Information

Figure S1 Cox-2 mRNA expression in WT (white bars) and Mmp9−/− (black bars) flexor tendons from three to 28 days post-repair. Data were normalized to *β-actin*, and WT day three expression. Data are presented as mean ± SEM.
(TIF)

Figure S2 Mmp9 mRNA expression in bone marrow from WT mice with Mmp9−/− bone marrow (white bar), and Mmp9−/− mice with WT bone marrow (black bar) at seven days post-repair. Data were normalized to *β-actin*, and expression in WT mice with Mmp9−/− bone marrow at seven days. (*) Indicates p<0.05, data are presented as mean ± SEM.
(TIF)
