# Supplementary figures and images for: Bone Marrow-Derived Matrix Metalloproteinase-9 Is Associated with Fibrous Adhesion Formation after Murine Flexor Tendon Injury
Source: PLoS One. 2012 Jul 11;7(7):e40602. doi: 10.1371/journal.pone.0040602 (PMC3394706; doi:10.1371/journal.pone.0040602)

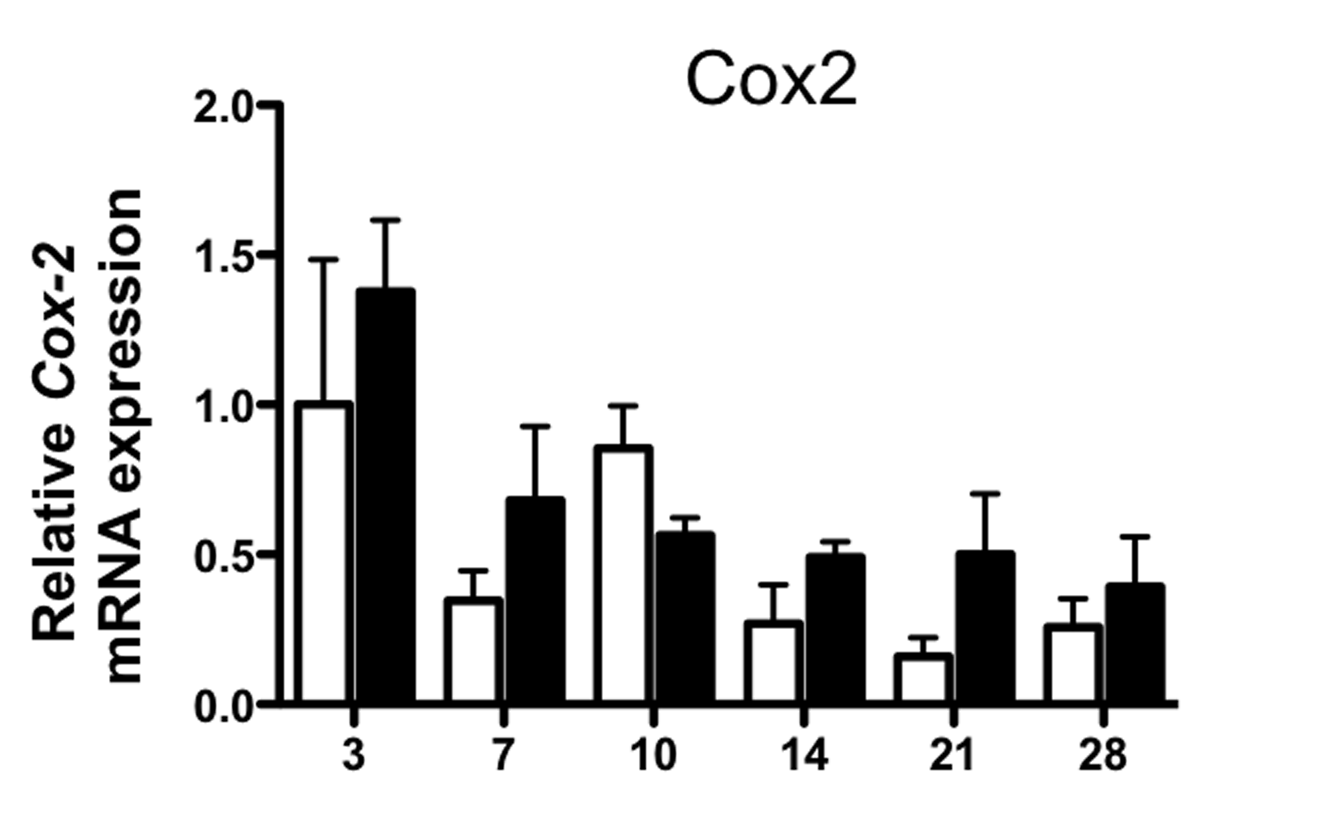

Supplement: Figure S1 — Cox-2 mRNA expression in WT (white bars) and Mmp9−/− (black bars) flexor tendons from three to 28 days post-repair. Data were normalized to β-actin, and WT day three expression. Data are presented as mean ± SEM. (TIF) [file pone.0040602.s001.tif]

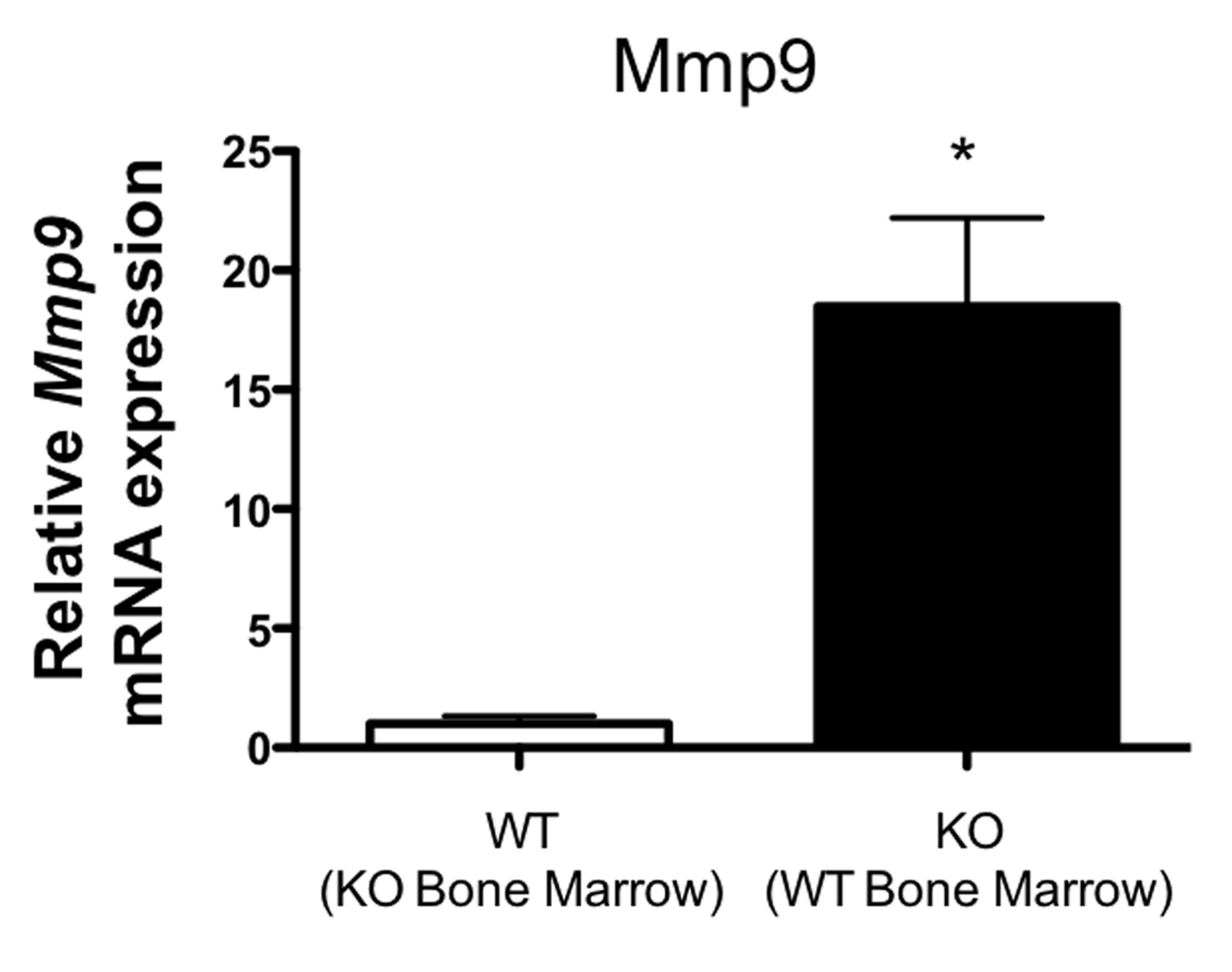

Supplement: Figure S2 — Mmp9 mRNA expression in bone marrow from WT mice with Mmp9−/− bone marrow (white bar), and Mmp9−/− mice with WT bone marrow (black bar) at seven days post-repair. Data were normalized to β-actin, and expression in WT mice with Mmp9−/− bone marrow at seven days. (*) Indicates p<0.05, data are presented as mean ± SEM. (TIF) [file pone.0040602.s002.tif]
